# *MYB*/*MYBL1*-altered gliomas frequently harbor truncations and non-productive fusions in the *MYB* and *MYBL1* genes

**DOI:** 10.1007/s00401-024-02803-0

**Published:** 2024-10-18

**Authors:** Hye-Jung Chung, Sharika Rajan, Zhichao Wu, Christina K. Ferrone, Mark Raffeld, Ina Lee, Jeffrey Gagan, Christopher Dampier, Zied Abdullaev, Manoj Tyagi, Patrick. J. Cimino, Martha Quezado, Kenneth Aldape

**Affiliations:** 1 Laboratory of Pathology, Center for Cancer Research, National Cancer Institute, National Institutes of Health, 10 Center Dr., Room 2S235, Bethesda, MD 20892 USA; 2 Surgical Neurology Branch, National Institute of Neurological Disorders and Stroke, Bethesda, MD 20892 USA

**Keywords:** *MYB*, *MYBL1*, Glioma

## Abstract

Astrocytomas that harbor recurrent genomic alterations in *MYB* or *MYBL1* are a group of Pediatric-type diffuse low-grade gliomas that were newly recognized in the 2021 WHO Classification of Tumors of the Central Nervous System. These tumors are described in the WHO classification as harboring fusions in *MYB* or *MYBL1*. In this report, we examine 14 consecutive cases in which a *MYB* or *MYBL1* alteration was identified, each with diagnostic confirmation by genome-wide DNA methylation profiling (6 Angiocentric gliomas and 8 Diffuse astrocytomas, *MYB-* or *MYBL1*-altered), for their specific genomic alterations in these genes. Using RNA sequencing, we find productive in-frame fusions of the *MYB* or *MYBL1* genes in only 5/14 cases. The remaining 9 cases show genomic alterations that result in truncation of the gene, without evidence of an in-frame fusion partner. Gene expression analysis showed overexpression of the *MYB*(*L1*) genes, regardless of the presence of a productive fusion. In addition, *QKI,* a recognized fusion partner common in angiocentric glioma, was generally up-regulated in these 14 cases, compared to a cohort comprising >1000 CNS tumors of various types, regardless of whether a genomic alteration in *QKI* was present. Overall, the results show that truncations, in the absence of a productive fusion, of the *MYB*(*L1*) genes can likely drive the tumors and have implications for the analysis and diagnosis of Angiocentric glioma and Diffuse astrocytoma, *MYB-* or *MYBL1*-altered, especially for cases that are tested on panels designed to focus on fusion detection.

**Supplementary Information:**

The online version contains supplementary material available at 10.1007/s00401-024-02803-0.

## Introduction

*MYB* (V-myb avian myeloblastosis viral oncogene homolog) proto-oncogene and *MYBL1* (V-myb avian myeloblastosis viral oncogene homolog-like 1) belong to a family of genes that encode transcription factor proteins regulating hematopoiesis and tumorigenesis [9]. *MYB* gene amplifications have been reported in hematopoietic malignancies, breast and colon cancers, whereas *MYBL1* alterations have been reported in Burkitt lymphoma [6]. Among CNS tumors, *MYB* or *MYBL1* alterations are noted in the current World Health Organization (WHO) classification as 2 tumor types: “Angiocentric glioma” and “Diffuse astrocytoma, *MYB*- or *MYBL1*-altered,” both as WHO grade 1 [1, 3, 7]. Angiocentric gliomas typically show alterations in the *MYB* gene, most often rearrangements with *QKI* as a partner [10], although other partners have been also described [3]. Conversely, Diffuse astrocytoma, *MYB*- or *MYBL1*-altered tumors show structural variations that result in a fusion involving more commonly *MYBL1*, although *MYB* alterations also occur. In both types, tumors are generally located in the supratentorial compartment and patients often present with refractory seizures [5, 14]. On imaging, both tumors are typically hypointense on T1 and hyperintense on T2 without enhancement [8]. At a practical level, diagnostic screening for *MYB* or *MYBL1* alteration often occurs at the level of relevant fusion detection, and the current WHO classification for Diffuse astrocytoma, *MYB*- or *MYBL1*-altered, emphasizes either the identification of a rearrangement involving these genes, or a DNA methylation profile aligned with diffuse astrocytoma, *MYB*- or *MYBL1*-altered, as important diagnostic criteria of these tumors [1]. In this report, we explore a series of 14 consecutively collected cases, obtained from the NCI CNS tumor clinical consultation service, where we examine *MYB*(*L1*) alterations in detail.

## Materials and methods

### Sample preparation and diagnostics

The use of human subject material was performed in accordance with the World Medical Association Declaration of Helsinki and with the approval of the participating Institutional Review Board (IRB). A waiver of informed patient consent was obtained in an IRB-approved protocol at the National Cancer Institute. Patient material and clinical data were prepared, and patients were diagnosed as previously described [15]. This study included cases from the Laboratory of Pathology clinical consult service at the National Cancer Institute (NCI), USA. Tissue histopathology was examined by experienced pathologists (MQ, PJC, KA) involved in the clinical diagnosis of these cases.

### DNA methylation profiling and analysis

Samples were processed as previously described [15]. Genomic DNA extracted from FFPE tumor tissue (250 ng each as the standard) was bisulfite-converted (EZ DNA Methylation Kit, Zymo Research), processed using the Infinium FFPE DNA Restore kit (Illumina), and assayed on Infinium MethylationEPIC or MethylationEPIC v2.0 array (Illumina), following the Infinium HD FFPE Methylation Assay automated protocol. Methylation data were processed using versions 11b6 [2] and 12b6 (https://www.molecularneuropathology.org/mnp/classifiers) of the DKFZ Heidelberg classifiers. Copy number variant (CNV) profiles were inferred using the R “conumee” package (http://bioconductor.org/packages/conumee/) as implemented in the classifier package.

### RNA exome sequencing and analysis

RNA was extracted from 5 μm sections of FFPE tumor tissue using RNeasy FFPE Tissue Kit or AllPrep DNA/RNA FFPE Kit (Qiagen). RNA Exome Next-Generation Sequencing (NGS) libraries were prepared from 100 ng of each RNA using the RNA Prep with Enrichment (L) Tagmentation kit with TruSeq RNA Exome Panel (Illumina). Final enriched libraries were sequenced on NextSeq 550Dx or NovaSeq 6000 (Illumina). After sequencing, the FASTQ files were aligned to the human reference genome hg19 (GRCh37) using the STAR aligner [4] to generate BAM files. The resulting BAM files were used by the Arriba tool [13] to predict fusion calls. The filtered fusions (VCF file) are uploaded to the QIAGEN Clinical Insight (QCI; Qiagen) for annotation, classification, and interpretation. All variants were manually reviewed and confirmed by visualizing the raw sequencing read alignments on the Integrative Genomics Viewer [12].

## Results

**Fig. 1:**
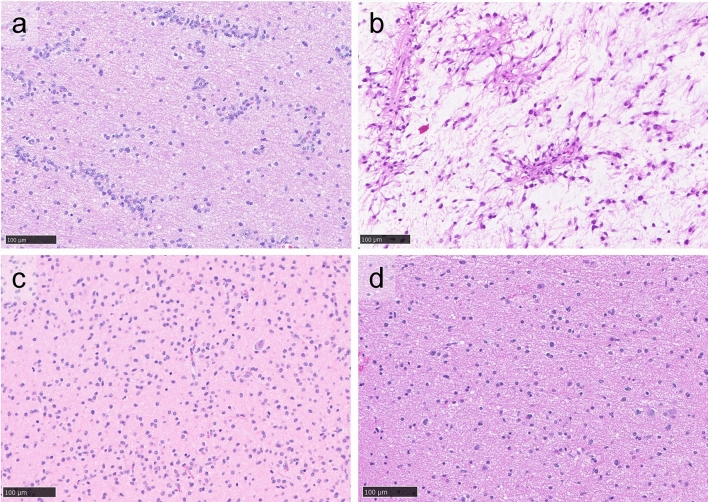
Histopathology of *MYB*(*L1*)-altered gliomas. Top: 2 cases of Angiocentric glioma (**a** Case 1; **b** Case 7), matching to methylation classes, LGG_MYB_D and LGG_MYB_B, respectively. Bottom: 2 cases of Diffuse astrocytoma, *MYB*- or *MYBL1*-altered (**c** Case 4; **d** Case 11), matching to methylation classes, LGG_MYB_C and LGG_MYB_D, respectively

Study cases included those which matched to the “Diffuse glioma, *MYB*- or *MYBL1*-altered” family by the CNS methylation classifier. In this cohort of 14 cases (Table 1 and Supplementary Table 1), the median age was 11 years (range 1–64) and the male-to-female ratio was 1.8 (9 males and 5 females). All but one were supratentorial, and the remaining case was located in the pons. A final diagnosis of angiocentric glioma was reached in 6 cases, and the remaining 8 cases were diagnosed as diffuse astrocytoma, *MYB-* or *MYBL1*-altered. With respect to specific methylation class, the DKFZ Heidelberg classifier divides this family into 4 classes, labeled Angiocentric glioma, *MYB*/*MYBL1*-altered (AG_MYB), and Diffuse astrocytoma, *MYB-* or *MYBL1*-altered, subtypes B, C, and D. In our cohort, 4 cases matched to the Angiocentric glioma, *MYB*/*MYBL1*-altered class, and all 4 were deemed to represent angiocentric gliomas in the final integrated diagnosis (Table 1). Interestingly, 2 additional cases (Cases 1 and 7), deemed to represent angiocentric glioma and showing the characteristic perivascular arrangement of tumor cells as well as positive results of EMA stains (Supplementary Table 1), matched to 2 of the other classes in the family (LGG_MYB_D and LGG_MYB_B, respectively; Fig. 1a, b). By comparison, 2 cases of diffuse astrocytoma, *MYB*- or *MYBL1*-altered (Cases 4 and 11), in which perivascular accumulation of tumor cells was not appreciated and which matched to the methylation classes, LGG_MYB_C and LGG_MYB_D, respectively, are also shown in Fig. 1c and d. The remaining 8 cases of diffuse astrocytoma, *MYB*- or *MYBL1*-altered all matched to the classes, Diffuse astrocytoma, *MYB*- or *MYBL1*-altered, subtypes B, C or D.

**Table 1:** Demographic and genomic characteristics of cohort

Case number	Age	Sex	Specimen location	Methylation class	RNA Sequencing result	RNA sequencing interpretation	Integrated diagnosis
Case 1	4	F	Right temporal	LGG_MYB_D	MYB-QKI	MYB in-frame fusion (to QKI)	Angiocentric glioma
Case 2	64	F	Left frontal	AG_MYB	MYB-QKI	MYB in-frame fusion (to QKI)	Angiocentric glioma
Case 3	2	M	Left parietal	LGG_MYB_D	MYB-PCDHGA1	MYB in-frame fusion (to PCDHG)	Diffuse astrocytoma, MYB- or MYBL1-altered
Case 4	8	M	Left parieto-occipital	LGG_MYB_C	MYB-PCDHGA1	MYB in-frame fusion (to PCDHG)	Diffuse astrocytoma, MYB- or MYBL1-altered
Case 5	18	M	Right temporal	AG_MYB	MYB-QKI	MYB truncation/non-productive fusion	Angiocentric glioma
Case 6	51	M	Left parietal	LGG_MYB_C	MMP16-MYB	MYB truncation/non-productive fusion	Diffuse astrocytoma, MYB- or MYBL1-altered
Case 7	21	M	Right frontal	LGG_MYB_B	MYB-Chr6:q26	MYB truncation/non-productive fusion	Angiocentric glioma
Case 8	36	M	Left pontine	LGG_MYB_B	MYB-Chr6:q26	MYB truncation/non-productive fusion	Diffuse astrocytoma, MYB- or MYBL1-altered
Case 9	9	F	Left temporal	AG_MYB	No fusion	MYB truncation/non-productive fusion	Angiocentric glioma
Case 10	1	M	Left frontal	LGG_MYB_D	MYBL1-MMP16	MYBL1 truncation/non-productive fusion	Diffuse astrocytoma, MYB- or MYBL1-altered
Case 11	3	M	Right parietal	LGG_MYB_D	MYBL1-MMP16	MYBL1 truncation/non-productive fusion	Diffuse astrocytoma, MYB- or MYBL1-altered
Case 12	6	M	Right insular	LGG_MYB_D	MYBL1-Chr8:q21.3	MYBL1 truncation/non-productive fusion	Diffuse astrocytoma, MYB- or MYBL1-altered
Case 13	19	F	Left temporal	AG_MYB	MYBL1-Chr6:q26	MYBL1 truncation/non-productive fusion	Angiocentric glioma
Case 14	14	F	Left parietal	LGG_MYB_C	MYBL1-KHDRBS3	MYBL1 in-frame fusion (to KHDRBS3)	Diffuse astrocytoma, MYB- or MYBL1-altered

**Fig. 2:**
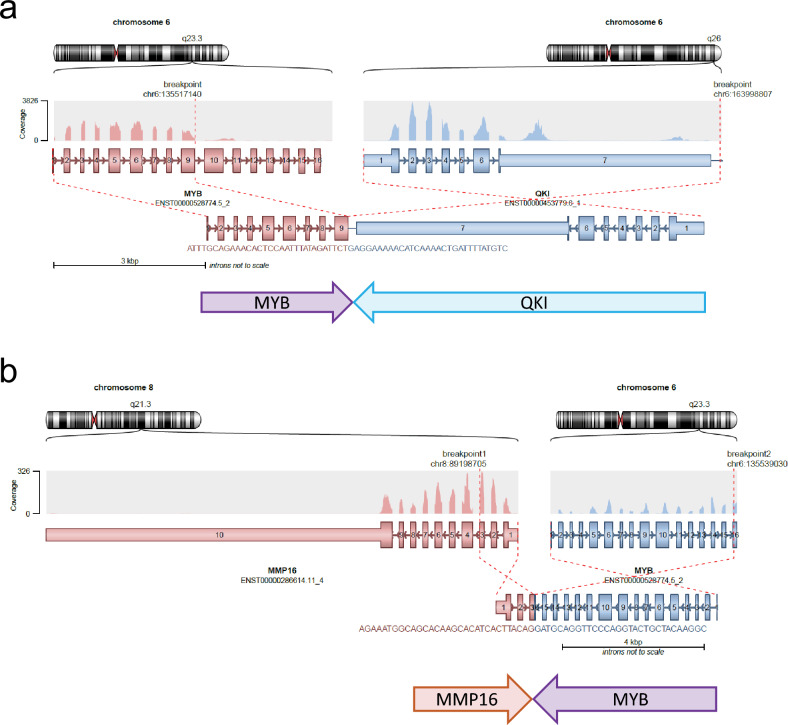
Tail-to-tail non-productive fusions in *MYB*/*MYBL1*-altered gliomas. Direction of each coding transcript is marked under the fusion diagram generated by Arriba. **a** *MYB*::*QKI* tail-to-tail rearrangement in Case 5. **b** *MYB*::*MMP16* (identified as *MMP16*::*MYB*) tail-to-tail rearrangement in Case 6

We then examined *MYB* and *MYBL1* alterations at the sequencing level using the TruSeq RNA Exome assay. *MYB*::*QKI* alterations were detected in 3 cases, but only 2 of them were in-frame fusions (Cases 1 and 2; Table 1) and the third one (Case 5) turned out having a tail-to-tail configuration of the fusion partner genes (Fig. 2a). Another type of in-frame *MYB* fusion involving the *PCDHG* gene cluster was found in 2 cases (Cases 3 and 4). Case 6 had a *MYB* fusion with a known partner, *MMP16*, but it was also a tail-to-tail fusion which cannot express a functional fusion protein (Fig. 2b). *MYB* was found to be rearranged to an intergenic region in chr6:q26 in 2 cases (Cases 7 and 8; Table 1).

**Fig. 3:**
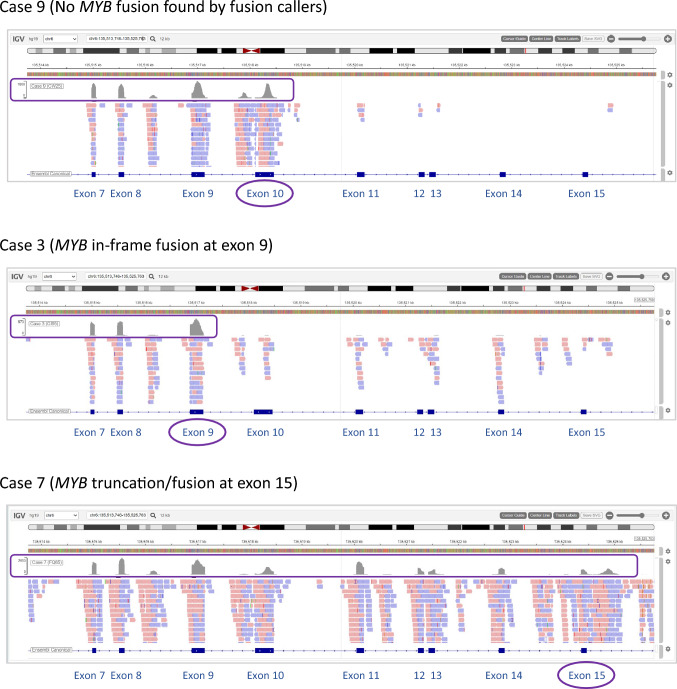
RNA sequencing coverage tracks of the *MYB* exons 7–15 region. Case 9 shows a reduction in read counts following exon 10, supporting a *MYB* rearrangement downstream of exon 10; Case 3 with a demonstrated in-frame *MYB* fusion at exon 9, shows a reduction in read counts following exon 9, representing a rearrangement downstream of exon 9; Case 7 with *MYB* truncation at exon 15, shows MYB read counts persistent up to exon 15

No *MYB*(*L1*) fusion or rearrangement was detected in Case 9 by 3 different fusion callers including Arriba, although this case had been classified to the “Diffuse glioma, *MYB*- or *MYBL1*-altered” family with maximum confidence scores (Supplementary Table 1). When the manual review of the sequencing reads didn’t reveal anything, either, we took on an unconventional approach of examining the overall sequencing coverage. The RNA sequencing coverage track of Case 9 for the *MYB* region showed abundant reads up to exon 10, and then a significant reduction to the background noise level following exon 10 (Fig. 3). We interpreted that this pattern supports the presence of a genomic rearrangement downstream of exon 10. By comparison, Case 3 with a *MYB* in-frame fusion at exon 9, as well as Case 7 with a *MYB* truncation at exon 15, were also examined for the same *MYB* exons 7 to 15 region (Fig. 3). The sequencing coverage track of Case 3 showed a reduction in read counts after exon 9, agreeing with the expected pattern. Case 7 coverage track displayed the *MYB* sequencing reads persisting up to exon 15, also complying with our interpretation. These illustrations demonstrate another means of rearrangement interpretation, even in the absence of an event detectable by standard NGS workflows.

Cases 10 and 11 had *MYBL1* rearrangements with the most common fusion partner, *MMP16*, however, both were out-of-frame fusions. Cases 12 and 13 had *MYBL1* rearranged to intergenic regions in chr8:q21.3 and chr6:q26, respectively.

Overall, among the 9 cases with *MYB* alterations, 4 were expected to result in productive fusions, with the remaining 5 alterations not expected to result in a productive fusion. Among the 5 cases with *MYBL1* alterations, only 1 was expected to result in a detectable in-frame *MYBL1* fusion (Case 14; *MYBL1*::*KHDRBS3*). The cases whose rearrangements were re-evaluated as being unable to produce in-frame fusion proteins, were collectively deemed “*MYB* truncation/non-productive fusion” or “*MYBL1* truncation/non-productive fusion” cases (Table 1).

**Fig. 4:**
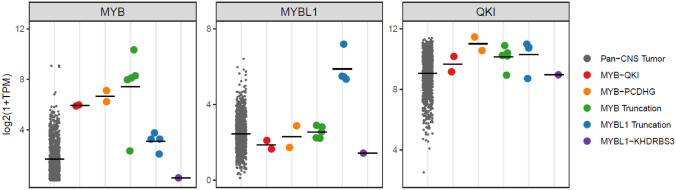
Expression levels of MYB, MYBL1 and QKI for each case in the cohort. Samples are sorted according to the type of fusion/rearrangement, and for comparison, the expression levels of 1005 pan-CNS tumor cases in the NCI clinical sequencing archives are also shown. The median level of each gene in each sample group is displayed as a line. TPM: transcripts per kilobase million

We then turned our attention to the expression of relevant genes in these tumors. Whether in-frame fusion or truncation, most *MYB*-rearrangement cases showed high levels of *MYB* expression, when compared within an archival cohort of  > 1000 CNS tumor cases profiled in the course of clinical testing on the same RNA Exome platform (Fig. 4). All 4 "*MYBL1* truncation/non-productive fusion" cases demonstrated high levels of *MYBL1* expression, but the singular in-frame fusion case did not. Interestingly, we found *QKI* expression to be generally elevated in all *MYB-* or *MYBL1*-altered glioma cases, relative to the pan-CNS tumor cohort, and not limited to those cases with genomic rearrangement positions found near the *QKI* gene (Fig. 4), prompting a speculation that this KH-domain-containing RNA-binding protein may play a role in the physiology of this glioma group, beyond the frequent fusion partner to *MYB*. We did not find a similar correlation in the expression patterns of other fusion partners.

## Discussion

In summary, our report of 14 cases in a defined methylation family, “Diffuse glioma, *MYB-* or *MYBL1*-altered” reveals several key issues: First, we note 2 cases of angiocentric glioma, which did not match to the specific angiocentric glioma subclass on methylation (Fig. 1a, b), highlighting the need to critically assess methylation profiling results within the context of the case in *MYB*/*MYBL1*-altered gliomas. Noting that histologically angiocentric patterns were identified in multiple methylation subclasses of *MYB*(*L1*)-altered gliomas, this finding could suggest that perivascular cell arrangements (and the *QKI* fusion partner) are not restricted to angiocentric gliomas, but potentially found in other methylation subclasses of the *MYB*(*L1*)-altered gliomas as well. Consequently, the distinction of angiocentric gliomas from what the WHO terms "diffuse astrocytomas, *MYB*/*MYBL1*-altered" could be revisited at the time of the next revision of the CNS WHO classification.

Second, the genetic alterations involving *MYB* or *MYBL1* are complex and frequently do not result in a productive in-frame fusion. These findings suggest that the presence of a truncated *MYB* or *MYBL1* gene, whether part of a productive fusion or not, is likely important in the molecular pathogenesis of these tumors. On a practical level, the results also suggest that testing for *MYB* or *MYBL1* fusions on many sequencing panels may not be sufficient to uncover the genomic alterations; indeed, in 5 of the cases that we received in consultation for methylation profiling, fusion testing had been performed at an outside institution, with the result of no detectable fusion identified (Supplementary Table 1), which likely prompted the request for methylation profiling. While our series of cases sent in consultation for methylation profiling may not be fully representative of *MYB*/*MYBL1*-altered gliomas in general, our results highlight a point at minimum that, if a productive fusion is not detected in a suspected case, a dedicated search should be considered for a possible “truncation/non-productive fusion” type of genomic alteration in *MYB* or *MYBL1*. Technical platforms feasible for such a search will not be limited to RNA sequencing, but also include DNA sequencing that utilizes NGS panels which cover certain introns of interest (as succeeded in the outside institution with Case 7; Supplementary Table 1).

The results from a recent report on 33 *MYB/MYBL1*-altered glioma cases show that a substantial proportion of the cases (at least 13/33) exhibited a rearrangement of the *MYB* or *MYBL1* gene in the absence of a recognizable fusion partner [8]. Prior functional genomics work showed that the truncated MYBL1 protein expression induced tumor formation in nude mice [11], supporting the notion that truncated *MYB*(*L1*) genes are a potential driver of these tumors. An additional finding of potential interest was the overall elevated levels of the *QKI* expression in the majority of the cohort, regardless of its involvement in *MYB* or *MYBL1* rearrangement (Fig. 4). Acknowledging that this is preliminary, we hope this finding may stimulate further work on the importance of this gene in *MYB*(*L1*)-altered tumors more broadly. While methylation profiling represents a robust means to identify these tumors, we note its limited availability at the current time. As such, NGS is likely to represent an important modality to screen for these alterations. It is hoped that our work can provide additional insights into the complexities of the rearrangements that are found in an effort to enhance the accurate diagnosis of these tumors.

In conclusion, our study elucidates the diverse set of rearrangements that occur in the *MYB* and *MYBL1* genes in *MYB*(*L1*)-altered glial neoplasms. While direct testing for productive in-frame fusions of these genes will define some cases of this group, our results show that truncation/non-productive fusions involving these genes are common and will often require a more thorough investigation in the molecular diagnosis of these tumors. Overall, further investigation of the various types of *MYB*/*MYBL1* alterations and their role in CNS tumor development is needed to precisely characterize these tumors and their clinical behavior.

## Supplementary Information

Below is the link to the electronic supplementary material.

Supplementary file1 (XLSX 15 KB)

## Data Availability

Processed methylation and genomic results and raw data are available upon reasonable request to the authors.

## Abbreviations

MYB: V-myb avian myeloblastosis viral oncogene homolog
MYBL1: V-myb avian myeloblastosis viral oncogene homolog-like 1
QKI: RNA-binding protein Quaking
PCDHG: Protocadherin Gamma Cluster
MMP16: Matrix Metallopeptidase 16
CNS: Central Nervous System
CNV: Copy Number Variant
DKFZ: Deutsches Krebsforschungszentrum
FFPE: Formalin-fixed paraffin-embedded
NGS: Next-generation sequencing
WHO: World Health Organization
IRB: Institutional Review Board
NCI: National Cancer Institute
BAM: Binary Alignment Map
VCF: Variant Cell Format
TPM: Transcripts Per kilobase Million
